# Raman spectroscopy for postmortem interval estimation of human skeletal remains: A scoping review

**DOI:** 10.1002/jbio.202300189

**Published:** 2023-08-09

**Authors:** C. Woess, Christian W. Huck, J. Badzoka, C. Kappacher, R. Arora, R. A. Lindtner, Philipp Zelger, M. Schirmer, W. Rabl, Johannes Pallua

**Affiliations:** ^1^ Institute of Forensic Medicine Medical University of Innsbruck Innsbruck Austria; ^2^ Institute of Analytical Chemistry and Radiochemistry University of Innsbruck Innsbruck Austria; ^3^ Department of Orthopaedics and Traumatology Medical University of Innsbruck Innsbruck Austria; ^4^ University Clinic for Hearing, Voice and Speech Disorders Medical University of Innsbruck Innsbruck Austria; ^5^ Department of Internal Medicine, Clinic II Medical University of Innsbruck Innsbruck Austria

**Keywords:** artificial intelligence, chemometrics, deep learning, human bone, orthogonal partial least squares regression, postmortem interval, principal component analysis, Raman parameters, Raman spectrometry

## Abstract

Estimating postmortem intervals (PMI) is crucial in forensic investigations, providing insights into criminal cases and determining the time of death. PMI estimation relies on expert experience and a combination of thanatological data and environmental factors but is prone to errors. The lack of reliable methods for assessing PMI in bones and soft tissues necessitates a better understanding of bone decomposition. Several research groups have shown promise in PMI estimation in skeletal remains but lack valid data for forensic cases. Current methods are costly, time‐consuming, and unreliable for PMIs over 5 years. Raman spectroscopy (RS) can potentially estimate PMI by studying chemical modifications in bones and teeth correlated with burial time. This review summarizes RS applications, highlighting its potential as an innovative, nondestructive, and fast technique for PMI estimation in forensic medicine.
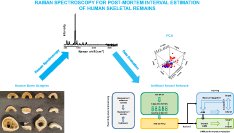

## Graphical abstract

Determining the postmortem interval (PMI) in forensic medicine is crucial for criminal investigations but challenging due to environmental factors affecting decomposition. Raman spectroscopy (RS) is a fast and objective method with valuable applications in estimating PMIs for forensic and anthropological analyses. This review emphasizes RS's significance in understanding bone decomposition and its contribution to analytical chemistry.

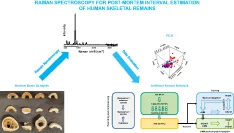

## 1 INTRODUCTION

Determining the postmortem interval (PMI) of human remains in medico‐legal contexts is often paramount, particularly concerning correct and speedy identification. More precisely, assessing the time of death is fundamental in forensic medicine as it may provide crucial insights into criminal cases. However, this procedure is one of the most complex forensic issues because of numerous environmental and endogenous factors involved in the corpse's decomposition [1, 2, 3]. Currently, after the discovery of skeletal remains, thanatological data are combined with information related to the place of discovery, in particular to the climatic and microclimatic conditions (temperature, humidity, rainfall, ventilation), to the geophysical and geochemical (soil type, pH, depth of burial) elements, and to the possible presence of fauna [4, 5, 6, 7, 8]. Thus, since PMI estimation considers the action of many—not always controllable—environmental factors, it is still peremptory to rely upon expert experience. Therefore, integrated and compared to each other under the critical eyes of experienced experts, many environmental and thanatological detections and observations can reduce error rates.

Consequently, in the case of dry bones, the forensic anthropologist can observe the aspect, color, weight, and odor and may be able to distinguish between forensic and archaeological remains. If both bones and soft tissues are still present, the forensic anthropologist can describe the remains' forensic pertinence without correctly estimating the time since death. The reason for this uncertainty is the lack of reliable methods to be applied. So far, several independent research groups have presented exciting and promising work on the PMI estimation issue in skeletal remains, but they did not provide valid data for forensic cases [8, 9, 10, 11, 12, 13, 14, 15].

Despite extensive research to objectively estimate the PMI for skeletal remains, the methods are expensive, time‐consuming, and unreliable for those with PMIs of more than 5 years. Therefore, a better understanding of bone decomposition, especially for assessing PMIs in skeletal remains of more than 5 years postmortem, is fundamental for forensic investigations. Therefore, estimating the PMI in fresh cadavers is usually not a problem, arising mainly in forensic anthropology cases [16, 17, 18]. Expanded data could be helpful also for archaeology and paleontology, focusing on the long‐term survival of bone or specific components, such as collagen, within the bone. Thus, the forensic field has proliferated as a branch of analytical chemistry [19]. Several studies focus on bones' chemical and physical changes after death and the factors affecting the diagenetic process [20, 21, 22]. So far, many methods have been studied, but they are still insufficient. Some of these methods are destructive, and others lack objectivity. Among these, Raman spectroscopy (RS) showed differences in the chemical composition of bones depending on the burial environment, with a coherent decline in organic Raman bands with the duration of the burial time [19]. Therefore, RS was used to evaluate the PMI by studying how far a bone's mass is related to the PMI [23]. Defleshed human ribs were used in various environments over 90 days. Raman spectra showed some “leveling” of peaks associated with organic components inside the bone within 3 months. The most notable changes were the broadening and decreasing of the amide I band, parallel with the CH_2_ region and the NH band. RS has demonstrated its application in determining the PMI of bones and teeth by studying their dynamic chemical modifications and correlating them with the time of burial [19, 24, 25]. Further advantages of RS are that it is microscopic, fast, and nondestructive (if the piece is small, to fit the equipment), which are of utmost importance in cases of forensic or anthropological analysis, in addition to the fact that there are also portable devices [19, 26, 27, 28].

This narrative review aims to summarize current literature on relevant technical types of equipment, including hardware and software and applications for RS as an innovative technique for PMI estimation in forensic medicine.

## 2 PHYSICAL BASICS OF RS

### 2.1 Infrared spectroscopy

Infrared spectroscopy is a method that has long been established for chemical analyses. The applications range from identifying various chemical substances to processing demanding scientific questions. The term “spectroscopy” includes matter interactions with electromagnetic radiation and is used today for different scientific issues, as shown in Figure 1. The infrared spectroscopy principle is based on the vibrational excitation of chemical bonds by radiation in the infrared range [29].

**FIGURE 1:**
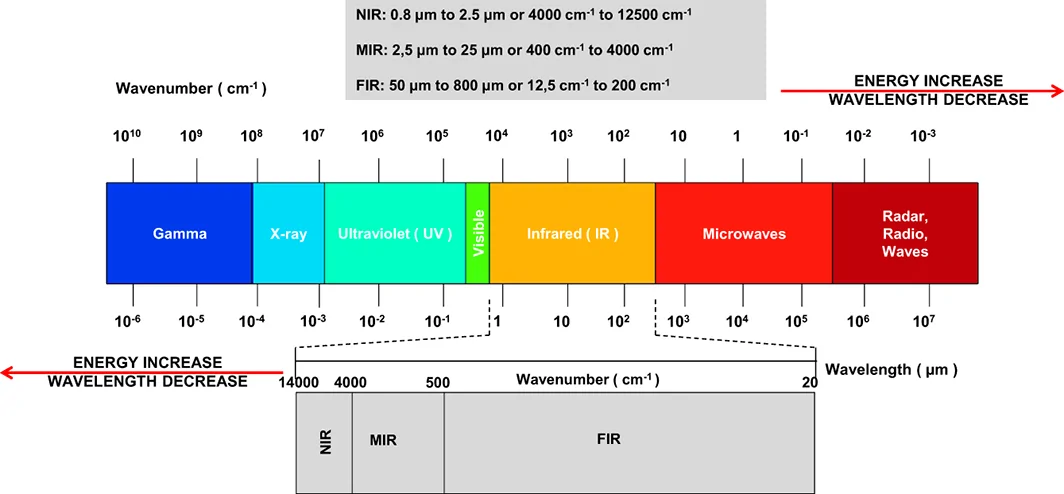
Schematic representation of the electromagnetic spectrum. Three IR spectral ranges detect changes in vibrational states: NIR, MIR, and FIR. The wavenumber is the common unit as cm^−1^ and is inversely proportional to the wavelength *λ*: *v* = 1/*λ* [30].

The energy of the molecular vibration is influenced by the size, the arrangement of the atoms involved and their bond strength. For this reason, infrared spectroscopy can be used to detect transitions between energy levels in molecules. These transitions result in stretching and bending vibrations of the interatomic bonds. The respective vibration frequency is characteristic of a specific functional group within the molecule. At room temperature, most molecules exist in their fundamental vibrational states. In order to excite molecules into a higher vibrational state, energy in the form of electromagnetic radiation must be absorbed. The reaction of the molecules depends on their molecular environment, sample morphology, and various physicochemical parameters. The physical principle of infrared spectroscopy is based on the absorption of incident electromagnetic radiation by various molecules, which converts them into defined excited vibrational and rotational states. When the change in vibrational and rotational states changes in dipole moment, absorption of infrared light occurs.

By detecting vibrational motions of functional molecular groups with simple or no sample preparation, vibrational spectroscopy techniques can extract a wealth of spectral information quickly and cost‐effectively [31, 32]. RS is a vibrational technique using a monochromatic light source, usually from a laser in the visible, near‐infrared, or near‐ultraviolet range, and is ideal for identifying skeletal remains [33, 34, 35, 36].

Indeed, fluorescence is a common problem in RS, and fluorescence can be a major problem because it can obscure and mask the RS signal, especially when examining biological samples such as unburned bones [37]. Fortunately, this concern can be solved by several techniques capable to reduce or eliminate fluorescence, such as using UV filters, laser wavelengths that minimize fluorescence, or fluorescence suppression algorithms that separate the RS signal from the fluorescence [38]. Thus, the different causes for the appearance of fluorescence in bone specimens like organic and inorganic components that tend to fluorescence when irradiated with light can be overcome (in particular, organic ingredients such as collagen and proteins are known to emit fluorescent signals [37]).

### 2.2 General aspects of RS

RS is an inelastic scattering technique involving an energy exchange between incident light and the molecules in the sample. This induces molecular vibrations. Light can be transmitted, absorbed, or scattered when it falls on a sample. Most electrons are scattered with the same energy and frequency as the incident light (= Rayleigh or elastic scattering). However, when an energy exchange occurs between the incident photons and the sample molecules, the frequency of the scattered light changes to that of the incident light. This induces molecular vibrations called Stokes Raman scattering [30].

When a photon falls on a molecule, energy is transferred in Stokes Raman scattering. The resulting scattered photon has lower energy than the incident photon. In anti‐Stokes Raman scattering, energy is transferred to the incident photon, and the scattered photon has higher energy than the incident photon. For anti‐Stokes Raman scattering to occur, the molecule must initially be at higher vibrational levels, which requires higher temperatures. Most molecules are in the fundamental vibrational state, so Stokes Raman scattering predominates at room temperature [39]. Figure 2 depicts the energy transitions for elastic (Rayleigh) and inelastic (Stokes) light scattering.

**FIGURE 2:**
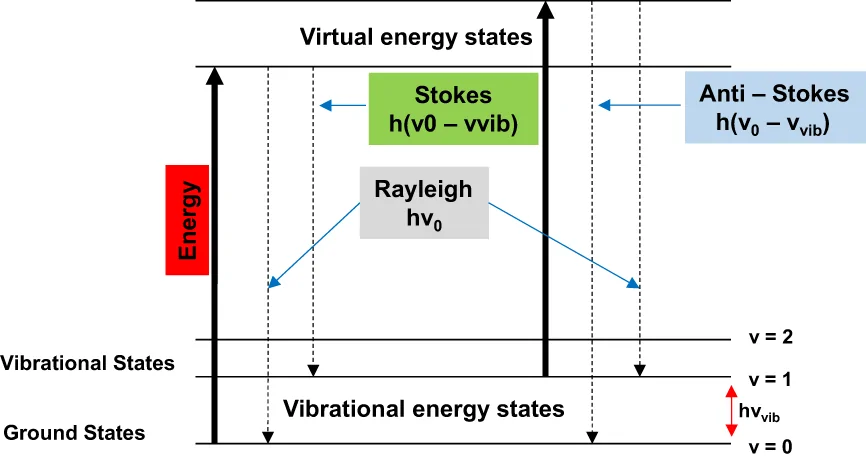
Energy transitions for Rayleigh and Stokes light scattering.

The molecule's vibrational frequency corresponds to the energy difference between excitation and scattered light. Each type of molecule has its molecular vibrations. Thus, the Raman spectrum consists of characteristic bands corresponding to that molecule's vibrational frequencies. The intensity of the Raman bands depends on the number of molecules or ions, which is why the technique can be used for quantitative analysis [40].

Raman spectra are molecular fingerprints that provide information about the sample's composition only through the presence of bands. Raman bands are sensitive to their immediate surroundings. Changes in these bands' peak position, intensities, and widths provide information about mechanical stress, temperature, bacteria's presence, and the material's structure [41]. Biological samples can also be measured in their typical environment [42]. Because the Raman spectrum of water is weak, it is possible to record spectra in aqueous solutions, which is impossible with MIR spectroscopy. For this reason, RS can provide complementary information about molecular vibrations and be used together [43].

Consequently, RS has become a versatile technique for analyzing bone tissue composition [26]. The Raman spectrum of skeletal remains provides extensive information about their minerals' and collagen's structure and composition and a complete examination of their diagenesis [28, 44, 45]. Analyses with RS are sensitive to changes in skeletal remains, making them feasible techniques for estimating the late PMI [19, 23, 46, 47]. Species discrimination via RS of osseous tissues has been previously investigated, but only fresh and intact bones were involved in these studies [48]. Decomposition, dissolution, mineral precipitation, and recrystallization can cause physical and chemical changes in skeletal remains over time [49].

## 3 RS FOR BONE MEASUREMENTS

There are two significant constituents of bones: the organic phase, which mainly consists of type I collagen fibers, and the inorganic (mineral) phase, a carbonated crystalline calcium phosphate closely equaling hydroxyapatite [50]. Various parameters can be determined from Raman spectra that are useful for the analysis of bone. Most of these can be calculated using peak heights (PHs) or by the area under the peaks at specific wavelengths. The assignment of Raman bands from bone measurements and the overall analysis is well established [51]. The same components discovered in bone IR spectra have been detected in Raman spectra. Vibration bands corresponding to phosphates, carbonates, organics such as amide I, II, and III, and CH‐aliphatics are among these components. Figure 3 shows a typical Raman spectrum of a human bone with a PMI of 0–2 weeks. Peaks at 427, 577, and 958 cm^−1^ are characteristic of phosphate (PO_4_^3−^). Peaks at 851, 873, and 917 cm^−1^ are counted for the collagen‐proline and hydroxyproline matrix, and the band at 1001 cm^−1^ is typical for phenylalanine. The Raman band at 1070 cm^−1^ is associated with carbonate bone minerals. The amide III Raman band, which is protein‐related, is located between 1210 and 1320 cm^−1^ and typically consists of two major components at 1246 and 1270 cm^−1^. These are assigned to the secondary structures of the collagen β‐sheet and the α‐helix. The peak at 1450 cm^−1^ is due to the CH_2_ deformation of the protein. The broad Raman band centered at 1656 cm^−1^ represents amide I of collagen, which typically consists of several secondary structures [52]. The CH stretching vibration can be found in the wavelength range of around 2800 to 3000 cm^−1^ [30].

**FIGURE 3:**
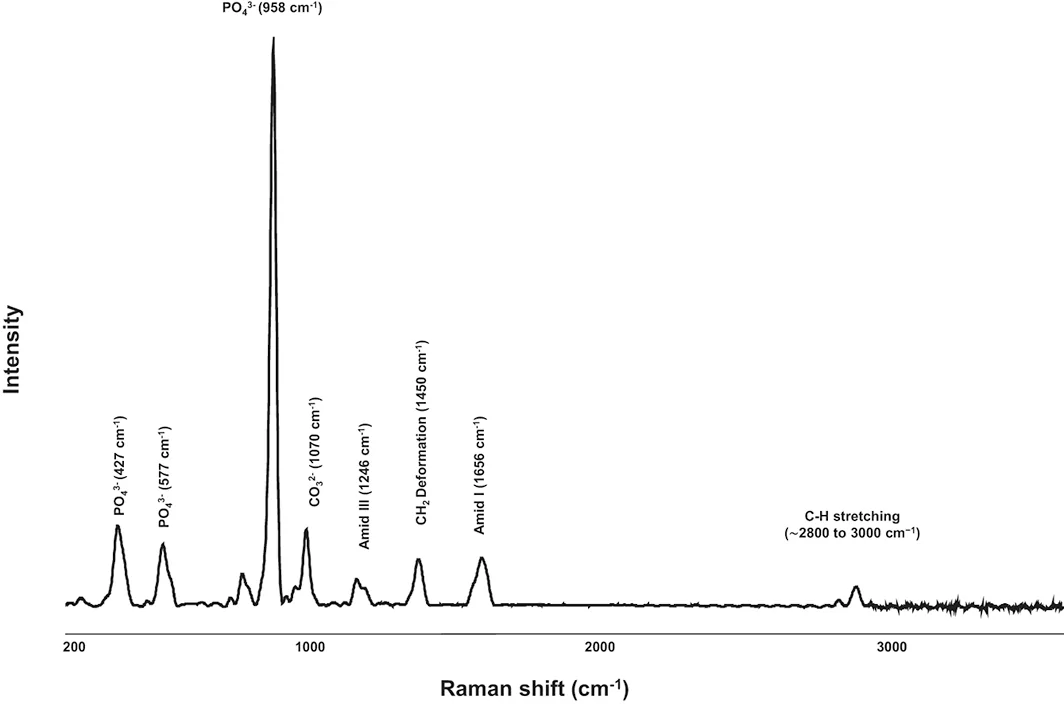
Raman spectrum of a human bone with a PMI of 0–2 weeks.

### 3.1 Raman parameters for bone analyses

Several Raman parameters can be applied to bone tissue. These are the crystallinity, mineral‐to‐mineral ratio, mineral‐to‐matrix (MMR) ratio, mineral carbonate content (MinCarb), mineral content (MinCont), carbonate‐to‐phosphate ratio, and collagen cross‐link ratio [53]. The Raman parameters described provided information on bone quality through composition and nanostructure. Quality includes the composition and architectural properties of the bone tissue. These features are essential to resist excessive deformation and breakage [54]. Bacteria can alter these bone quality parameters in mechanically compromised tissue or infection. RS can detect these changes and provides information about bone health and quality [54]. Therefore, it is a valuable technique for studying the mineral and organic phases of normal and altered bone tissue. The main analyzed parameters are summarized in Table 1.

**TABLE 1:** Raman‐derived spectral markers for the characterization of human bone

Name	Description	Determination	References
Phosphate	ν_1_PO_4_^3−^ Amount of phosphate	(I_958_)	[19, 51, 55, 56, 57, 58, 59, 60]
Crystallinity	Crystallinity Degree of order within the hydroxyapatite mineral crystals	1/FWHM_958_	[61]
MMR phosphate/amide I	ν_1_PO_4_^3−^/amide I Amount of the mineral component to the organic one	(A_958_/A_1656_)	[52, 61, 62]
Mineral quality and crystallinity carbonate/phosphate	ν_1_CO_3_^2−^/ν_1_PO_4_^3−^ The extent of carbonate incorporation in the hydroxyapatite lattice	(A_1070_/A_958_)	[61, 62]
MinCarb	v^1^CO_3_^2−^/(C—H) bend; CH_2_ wag	(A_1070_/A_1450_)	[51, 55]
MMR	Proline + hydroxyproline + proline/amide I Changes in the collagen matrix	I_851 + 873 + 917_/I_1656_)	[52, 63]
	Amide I Secondary bands Alterations in the collagen network	(A_1656_/A_1684_)	[30, 52]
Amide I	Amide I of α‐helical structures Structural organization and relative amount of collagen	(I_1656_)	[61, 64]
CHACont	CH_2_ stretching	(I_2940_)	[19, 56, 57, 58]

Abbreviations: A, area of the band; CHACont, CH‐aliphatic content; FWHM, full width at half maximum; I, intensity of the band; MinCarb, mineral carbonate content; MMR, mineral‐to‐matrix.

#### 3.1.1 Crystallinity

Crystallinity is a complex result. It reflects crystal size as well as chemical composition and structural order. When disordered calcium phosphate precipitates, crystals nucleate on collagen fibers. Over time, these precipitates become more extensive and structured, forming a plate‐like, hydroxyapatite‐like carbonate‐substituted calcium phosphate. Crystallinity correlates positively with crystal size, negatively with carbonate content, and increases with tissue age [65].

#### 3.1.2 Mineral‐to‐matrix ratio

The MMR is a MinCont marker and correlates with ash weight [30]. Ash weight measures the degree of mineralization in bone tissue. Heating bones obtain it to extreme temperatures, vaporizing water and destroying organic matter. Bone aging is associated with increased MinCont, which is reflected in an increase in the MMR [66, 67]. Thus, the MMR is a measure that indicates the mineralization in the bone and thus provides information about the composition of the bone tissue. This is reported as the ratio of the mineral‐specific Raman band intensities to the amide I band intensity or the phosphate band intensity to the calculated total intensity of the proline and hydroxyproline Raman bands. The MMR calculated by the amide I band is specific to the collagen matrix. The MMR from proline and hydroxyproline is individual for the changes in the collagen matrix [52].

#### 3.1.3 Carbonate‐to‐phosphate ratio

The carbonate‐to‐phosphate ratio measures the extent of carbonate substitution into the mineral crystal lattice. It is indirectly related to the carbon content measured by coulometry and elemental analysis.

The incorporation of carbonate into the crystal lattice occurs over time in two ways:

- by the growth of the mineral crystal;
- by conversion of carbonate to phosphate, hydroxide, and unstable sites in the mineral crystal lattice in existing minerals [65].

#### 3.1.4 MinCarb content

The MinCarb is another commonly used parameter in addition to the CI. It is based on the absorbance ratio of a carbonate peak divided by a phosphate peak. This, therefore, measures the ratio of carbonate to phosphate in bone. IR spectra can be used to identify some carbonate and phosphate peaks. Therefore, many different forms of MinCarb have been used by the following authors: Nagy et al. [36], Patonai et al. [34], and Longato et al. [35] used the carbonate peak at 1428 cm^−1^ and the υυ_1_, υυ_3_PO_4_^3−^ phosphate peak at about 1042 cm^−1^. Thompson et al. [68, 69], Chadefaux et al. [70], Squires et al. [71], Howes et al. [46], Nielsen‐Marsh and Hedges [72, 73], Smith et al. [74], and Wright and Schwarcz [75] used the type B carbonate peak at 1415 cm^−1^ and the υυ_1_, υυ_3_PO_4_^3−^ phosphate peak at 1035 cm^−1^, while Lebon et al. [76] also used the 1415 cm^−1^ carbonate peak but measured the υυ_1_, υυ_3_PO_4_^3−^ phosphate peak at 1045 cm^−1^. Alvarez‐Lloret et al. [77] used a carbonate peak at 1405 cm^−1^ and used the maximum of the υυ_1_, υυ_3_PO_4_^3−^ phosphate peak without designating a specific wavenumber. Petra et al. [78] and Paschaslis et al. [79] also did not designate a specific wavenumber but vibrational bands and used the υυ_2_CO_3_^2−^ (carbonate) and the υυ_1_, υυ_3_PO_4_^3−^ (phosphate bands). Trueman et al. [80] chose a different approach to calculate the MinCarb and instead used the 1415 cm^−1^ carbonate (designating this peak as type B carbonate) and the carbonate peak at 1540 cm^−1^ (type A carbonate), and instead of using υυ_1_, υυ_3_PO_4_^3−^ phosphate, they used υυ_4_PO_4_^3−^ at 605 cm^−1^ as the phosphate measurement. Grunenwald et al. [81] tried to find out which method was best to estimate MinCarb. The research results of Grunenwald et al. [81] showed that using the υυ_3_CO_3_^2−^ (carbonate peak centered at about 1415 cm^−1^) and the υυ1, υυ_3_PO_4_^3−^ vibration (the phosphate peak centered around 1040 cm^−1^) were the most suitable. Grunenwald et al. [81] explored whether using peak area (PA) or PH gave better results. The study found that although the use of PH provided adequate results, they were lower than the results obtained with PA. Another measurement commonly used by researchers is the ratio of bone mineral (phosphate) to bone organic matrix. This is referred to as the MinCont ratio. This is measured by the ratio of the phosphate band of υυ_1_, υυ_3_PO_4_^3−^ to the amide I band (1580–1705 cm^−1^), with absorption peaks typically used between 1640 and 1660 cm^−1^ [46, 70, 78, 79, 82, 83]. In addition to these standard indices, Alvarez‐Lloret et al. [77] mention CH aliphatics present around 2900–2700 cm^−1^. It is also possible to establish a relationship between the CH aliphatics and the υυ_1_, υυ_3_PO_4_^3−^ phosphate band between 900 and 1200 cm^−1^ [24].

#### 3.1.5 Mineral content

For the MinCont, the shift between the mineral (phosphate) and organic (amide I) content of bone is measured. In IR and RS, the amide I band results from the stretching vibration of the peptide carbonyl group (—C=O). The existence of the amide I band in spectra is predominantly because of the presence of collagen [84, 85]. On Raman spectra of bone, the amide I band is at 1668 cm^−1^ [64]. For the calculation of MinCont from Raman spectra, the 1668 cm^−1^ amide I band and the υυ_1_PO_4_^3−^ phosphate band at 960 cm^−1^ is applied. France et al. [86] used RS and postulated that the 1636 cm^−1^ amide I peak was preferable to use compared to the 1450 cm^−1^ carbonate peak (MinCarb) or the 1668 cm^−1^ amide I peak, although these peaks also showed a high coherence. Regarding the RS MinCarb, Pestle et al. [60] also used the 1638 cm^−1^ amide I peak like France et al.'s [86]. Contrary to this, the MinCont ratio was reversed: 1638 cm^−1^/960 cm^−1^. Thus, the results showed the opposite, mainly that the MinCarb (1450 cm^−1^/960 cm^−1^) ratio correlated better with bone collagen content than the MinCont. The explanation may be the reversal of the standard MinCont ratio where the phosphate (inorganic) is divided by the amide I (organic) instead of (organic/inorganic) used by Pestle et al. [60].

#### 3.1.6 Collagen‐crosslinking ratio

The collagen cross‐link ratio is calculated using the ratio of the amide I subbands. This ratio indicates the maturity of the collagen fibrils and the extent of mature cross‐linking. This allows conclusions to be drawn about disturbances in the secondary structure of the collagen [87].

#### 3.1.7 CH‐aliphatic content

Another opportunity to quantify the organic to inorganic components within the bone is the CH‐aliphatic content (CHACont). Typically, the CH‐Aliphatic peak is related to proteins and lipids within the bone [58]. Maybe because the aliphatic vibration band declines quickly over time, the ratio is not largely utilized in IR and RS studies.

For receiving the ratio, the CH‐aliphatic vibration is generally at or around 2940 cm^−1^ in Raman spectra of bone and is divided by the υυ_1_PO_4_^3−^ phosphate band at 960 cm^−1^. The CHACont is partly calculated utilizing relative intensities of the peaks (PH) or the PA. The CHACont has been used for archaeological studies [56], in forensic science [19], and also for species differentiation [57]. Halcrow et al. [88] tried to assess the survival of bone protein in archaeological bones.

### 3.2 Chemometrics

Despite using RS and other analytical techniques in determining the PMI, few research studies have applied them in combination with chemometrics. Chemometrics transforms the recorded data into more valuable and interpretable information with which a predictive model can be built. This predictive model relates several variables or parameters, allowing the examination of the hidden information in a set of data associated with each other but influenced by numerous variables. These chemometric treatments extract helpful information from the trends in the multivariate data set [25, 26, 35, 47].

#### 3.2.1 Principal component analysis

The principal component analysis (PCA) is a multivariate statistical method to filter out the essential information from experimental data. Mathematical and statistical methods are used for this. The aim is to classify and reduce the data set's dimensions while retaining as much variation as possible in the original variables [89]. Therefore, the data are initially scaled to homogenize the scattering of the objects. Additionally, they are mean‐centered to improve the interpretability of the model. Most of the time, only the first main components are taken into account in the PCA, and the rest is neglected. The first principal component (principle component = PC 1) indicates the direction of the maximum variance of the projected data. Each object is projected onto PC 1 to get a coordinate value along the PC line. A PC is generally insufficient to describe a data set's variation. A second principal component (PC 2) is introduced the second‐largest data variation source. PC 1 and PC 2 define a model level in variable space. The coordinate values of the objects on this level are called scores. The PC loadings express the orientation of the model level in the variable space (see Figure 4) [90].

**FIGURE 4:**
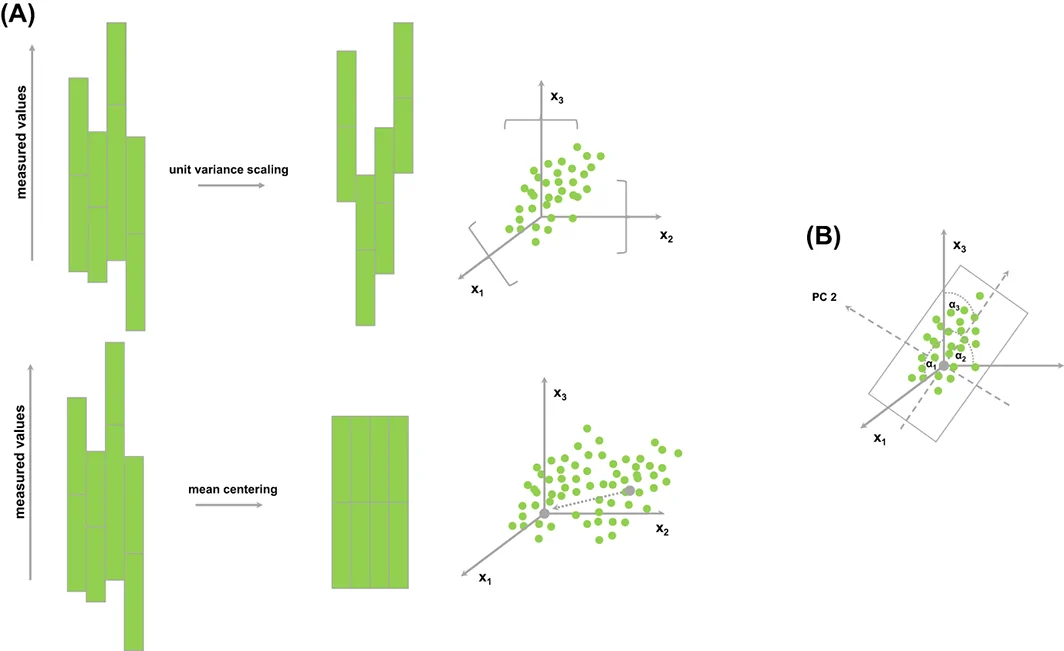
(A) Schematic representation of two standard preprocessing methods used at the beginning of a principal component analysis (PCA) (unit variance scaling and mean centering). (B) Graphical representation of the first two principal components that define a model plane in variable space.

#### 3.2.2 Orthogonal partial least squares regression

The combination of RS with orthogonal partial least squares regression (OPLSR) has proved to overcome the inefficiency of single analytical techniques in the study of the diagenetic processes to which human skeletal remains are subjected during burial by enabling the visualization and interpretation of the modifications experienced in the spectra of human skeletons according to their PMI. Despite a previous exploratory analysis for the detection of outliers and a preprocessing of the analytical signals, the significant interindividual variability of the skeletons and the large number of external and internal factors that influence their diagenesis have been decisive to fail in the construction of an OPLS model capable of estimating with acceptable accuracy the PMI of all the samples in the validation set. In conclusion, the nondestructive and confirmatory nature, versatility, portability, selectivity, accuracy, and the ability to provide an estimated error associated with the result provided via chemometrics make RS, in combination with the OPLSR method a promising technique for determining the PMI in forensic settings where only human skeletal remains are available for analysis [28].

### 3.3 Artificial intelligence

Artificial intelligence (AI) is rapidly advancing and involves the simulation of problem‐solving and decision‐making tasks that require human intelligence. Learning from experience is a crucial aspect of human intelligence, and machine learning (ML) is a subset of AI that addresses this ability, enabling machines and software to learn from experience. While AI and ML are closely related, ML is a distinct subset of AI [91]. Through classification techniques, ML can enhance outcome prediction by sorting data into predetermined categories. Both AI and ML are emerging fields with growing applications in forensic medicine [92], including forensic anthropology [93], forensic psychiatry [94], forensic odontology [95], postmortem imaging [96, 97], and PMI estimation [98]. AI has been used in various forensic research, including face recognition [99], cybercrimes [100], age inference [101], cause of death analysis [102], and gene and protein profiling [103, 104]. It was also used to evaluate the suitability of handheld near‐infrared spectrometry to estimate the PMI of human bones. The deep‐learning approach was used to correlate the PMI with the detected spectral properties. The study found that handheld NIR spectrometry could distinguish between forensic and archaeological bone material. The ML approach allows for extracting intrinsic information from spectra without knowing what information one is looking for. The neural network learns the features of the data and extracts the required information to form a decision on the age classes. The positive results of this study indicate that this method is accurate in differentiating between skeletal remains with a PMI of less than 10 years and historical samples older than 100 years. However, the study was limited to samples with a PMI between 0 and 10 years. Further research is needed to improve the accuracy of IR spectrometry in estimating PMI, especially during the first days [105]. A schematic illustration of a possible IR AI PMI workflow is presented in Figure 5. The first step involves recording and preprocessing by measuring reference spectra, spectra, and calibration. Next, the dataset is divided into age classes, and the labeled spectra are split into a training and test set based on their age classes. The training set is used to refine and train the artificial neural networks, while the test set evaluates its predictive abilities.

**FIGURE 5:**
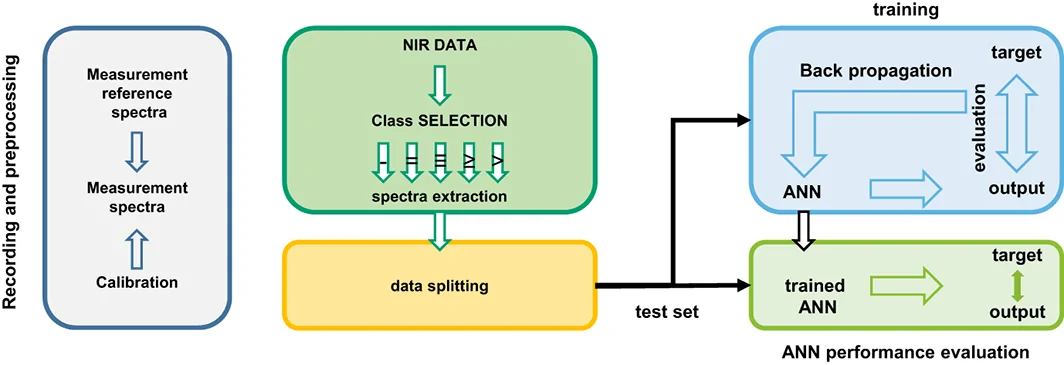
A schematic diagram of the study approach is presented. The methodology involved utilizing a deep‐learning‐based model, which applies artificial neural networks (ANNs) to extract hyperspectral NIR information.

## 4 RS OF SKELETAL REMAINS

The composition of synthetic hydroxyapatites applied in reconstruction surgeries as a surrogate for bone [106] was analyzed, as well as the impact of fluoride treatment on bones [107]. The variations in bone concerning the biological age were also studied [108]. Furthermore, RS was used to estimate carbonate and phosphate vibrational bands [55, 59]. A possible workflow for RS for PMI estimation is depicted in Figure 6.

**FIGURE 6:**
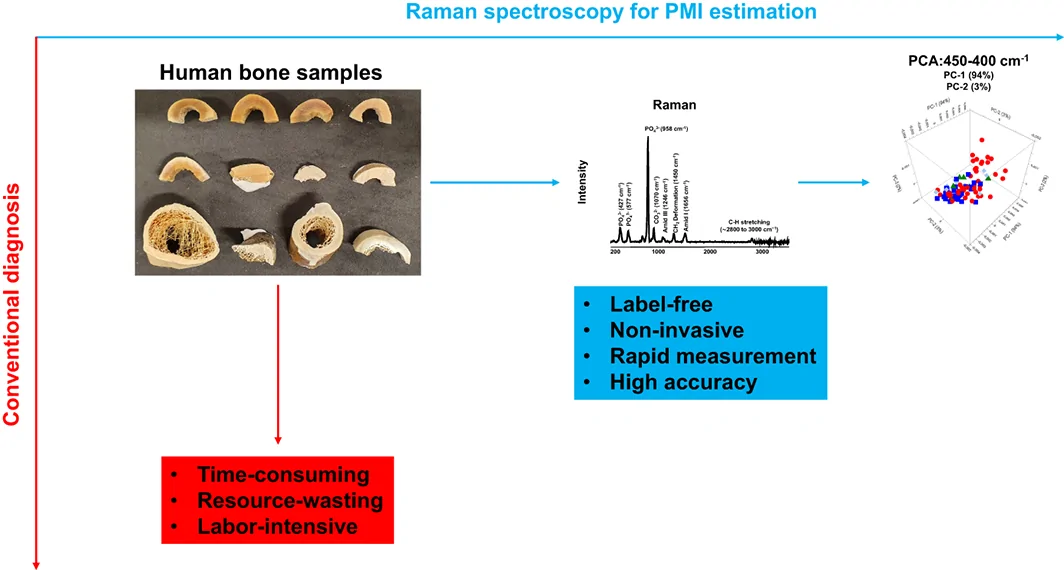
Workflow for Raman spectroscopy (RS) for postmortem interval (PMI) estimation.

### 4.1 RS and species determination

The species identification of a particular bone is also interesting in forensics [109]. McLaughlin and Lednev reasoned that with RS, it is possible to differentiate bones from four species (chicken, turkey, pig, and cow). They also postulated that further research regarding the impact of specimen age, bone type, and gender would be necessary. Edwards et al. [57, 110] used Fourier transform RS (FT‐RS) to define variations in ivories of distinct species by the usage of the ratio of organic (at 2930 cm^−1^) to inorganic (960 cm^−1^) peaks. FT‐RS was also used by Brody et al. [111] to differentiate ivory from the African elephant, mammoth, sperm whale, walrus, and hippopotamus. With the application of principal components analysis, discrimination between them was possible, but the graduation of each species was not 100% due to an intersection between some groups.

### 4.2 RS and collagen preservation

As with some prescreening, RS is useful for determining collagen in archaeological bones because collagen is utilized for radiocarbon dating, isotopic analyses, and DNA analyses. France et al. [86] used FT‐RS and postulated visual differences in the spectra of bones with poorly or well‐preserved collagen. In practice, the well‐preserved samples showed increased organic peaks connoting amide III, amide I, CH_2_, and CH regions compared to poorly preserved samples. After preceding experiments with four different handheld spectrometers, Pestle et al. [60] postulated an assessment of bone collagen to identify the suitability of bones for further analysis by applying the 1450 cm^−1^ carbonate peak and 960 cm^−1^ phosphate PH ratio (MinCarb). He also reasoned that the MinCarb (1450 to 960 cm^−1^) is a preferable diagnostic value for prescreening of collagen in bones. Halcrow et al. [88] found a high coherence between the CHACont (2940 cm^−1^/960 cm^−1^) and the collagen, carbon, and nitrogen contents of bone. Thus, RS is a helpful tool for evaluating the quantity and quality of collagen within the archaeological bone.

## 5 DISCUSSION

Over the past years, multiple studies have been conducted to explore the use of vibrational spectroscopy in analyzing bones for forensic purposes [112]. These studies have examined various questions, such as comparing analytical techniques [35, 113] and chemometric methods [47], estimating the PMI [28, 114], and differentiating between archaeological and forensic bones [105, 115]. While most of these studies have used infrared spectroscopy, Raman microspectroscopy has been utilized to a lesser extent and may have been underestimated. Indeed, vibrational techniques have a high potential in forensic investigations, but some challenges still need to be addressed before they can be fully utilized. One of the main issues is the impact of intrinsic and extrinsic factors on the spectra of bones, which may lead to a lack of precision and reproducibility. Previous studies have attempted to address this issue using chemometric techniques, such as PCA and partial least squares regression, but limitations still exist. This review highlighted the advantages of RS as a label‐free, noninvasive technique with high accuracy and rapid measurement. Since there is only a handful of publications from the last 10 years regarding the estimation of the PMI of skeletal remains available, there is a lot of scientific work to do to improve the estimation of the PMI of skeletal remains. A precise method for determining the PMI of skeletal remains must differentiate between recent and archaeological remains. The lack of accurate methods that permit assessment of PMI estimation in skeletal remains further urges forensic anthropologists and pathologists to continue deepening their knowledge of physical methods already established in the forensic field as it has been applied to drugs [116], hair [117], identifying ivory [57]. RS is also used in medical research [107, 108] or in the monitorization of protein structure in muscle food systems [118]. Both were investigating promising reliable methods like RS and sharing data on forensic cases by experts worldwide will provide validated new tools to be recommended as forensic methods.

## 6 FUTURE PERSPECTIVES

Future studies should aim to critically evaluate the use of RS in estimating the burial time of bones, considering the effects of intrinsic and extrinsic factors. Several essential aspects require additional work: (1) the introduction of calibration standards, (2) the development of technical and scientific protocols, and (3) improving our understanding of the origin and change of spectral absorption features. In summary, the following points must be addressed for future PMI research using RS:

- Optimization of data analysis with the development of technical and scientific protocols.
- Establishment of calibration standards and validation of techniques.
- Development of RS handheld units.
- Evaluation of possible legal consequences of RS use in prospective multicenter studies.

Finally, combining information from light microscopy, proteomics, metabolomics, RNomics, genomics, and radiology with RS data will improve the interpretation of vibrational spectra collected from bone samples and skeletal remains to assess the PMI for forensic and anthropological purposes.

## CONFLICT OF INTEREST STATEMENT

The authors declare no conflict of interest. The data presented in this review are available on request from the corresponding author.
